# Novel Cyano‐Artemisinin Dimer ZQJ29 Targets PARP1 to Induce Ferroptosis in Pancreatic Cancer Treatment

**DOI:** 10.1002/advs.202501935

**Published:** 2025-05-19

**Authors:** Jianping Chen, Lingyun Yue, Yanna Pan, Bingying Jiang, Junfeng Wan, Haixia Lin, Fujiang Guo, Huiyu Li, Yajuan Li, Qingjie Zhao

**Affiliations:** ^1^ State Key Laboratory of Discovery and Utilization of Functional Components in Traditional Chinese Medicine Shanghai Frontiers Science Center for TCM Chemical Biology Innovation Research Institute of Traditional Chinese Medicine Shanghai University of Traditional Chinese Medicine Shanghai 201203 China; ^2^ Institute of Interdisciplinary Integrative Medicine Research Shanghai University of Traditional Chinese Medicine Shanghai 201203 China; ^3^ Department of Chemistry College of Sciences Shanghai University Shanghai 200444 China; ^4^ School of Pharmacy Shanghai University of Traditional Chinese Medicine Shanghai 201203 China; ^5^ College of Mathematics and Physics Shanghai University of Electric Power Shanghai 201306 China

**Keywords:** anti‐pancreatic cancer, ferroptosis, novel artemisinin derivatives, PARP1 inhibitor

## Abstract

Pancreatic cancer remains one of the most lethal malignancies in the digestive system, with limited available drugs and a need for improved efficacy. This unmet clinical need highlights the urgency to discover novel, highly efficient small‐molecule compounds. Herein, a novel cyano‐containing artemisinin dimer derivative, ZQJ29, is synthesized through structural modifications of artemisinin. Biological evaluation demonstrated that ZQJ29 effectively inhibits the proliferation of pancreatic cancer cells both in vitro and in vivo. ZQJ29 selectively targets PARP1 and has distinct structural features comparable to established PARP1 inhibitors such as Olaparib. Notably, ZQJ29 is the first reported artemisinin derivative to inhibit PARP1. Furthermore, the inhibition of PARP1 by ZQJ29 enhances the expression of TP53 and inhibits the SLC7A11/GPX4 pathway. The work first demonstrates that targeting PARP1 can induce ferroptosis in pancreatic cancer. These findings not only identify promising artemisinin derivatives for the development of therapies targeting pancreatic cancer but also provide scientific evidence supporting therapeutic strategies aimed at inducing ferroptosis in pancreatic cancer. This research lays a robust foundation for subsequent preclinical studies.

## Graphical abstract

A novel artemisinin derivative dimer has been synthesized, and its significant potential as an anti‐cancer agent. The study indicated that ZQJ29 effectively inhibited the proliferation of pancreatic cancer cells in vitro and in vivo. Additionally, these investigations have unveiled ZQJ29 as a new potent PARP1 inhibitor, with the capability to induce ferroptosis in pancreatic cancer cells.

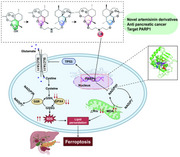

## 1 Introduction

It is well known that cancer is a clinically hazardous disease with a very high mortality rate.^[^ ^1^ ^]^ Within that existing category, pancreatic cancer is one of the common malignant tumors in the digestive system with increasing morbidity in recent years.^[^ ^2^ ^]^ Pancreatic cancer is usually characterized by late diagnosis, early metastasis, poor prognosis, and high recurrence rates, as well as the lowest 5‐year survival rate among all malignant tumors.^[^ ^3^ ^]^ Therefore, surgical resection remains the sole potentially curative treatment for pancreatic cancer, while few patients meet the indications for surgery.^[^ ^4^ ^]^ Gemcitabine‐based chemotherapy remains the first‐line clinical regimen for pancreatic cancer; clinical issues such as poor targeting, high toxicity, significant side effects, and drug resistance persist.^[^ ^5^ ^]^ On the other hand, immunotherapies, including cytokine therapy, immune checkpoint inhibitors, and chimeric antigen receptor T‐Cell therapy, have demonstrated minimal efficacy in treating pancreatic cancer.^[^ ^6^ ^]^ In light of these challenges, the immediate priority for the medical community is to discover new drugs that enhance anti‐tumor responses, improve patient outcomes, and ultimately elevate survival rates. Natural products with diverse chemical structures and various biological activities play a crucial role in drug discovery. Numerous studies have highlighted the anti‐pancreatic cancer effects of natural products through diverse molecular mechanisms.^[^ ^7^ ^]^

Poly(ADP‐ribose) polymerases (PARPs) are a class of multifunctional protein post‐translational modification enzymes widely found in eukaryotic cells, which play important roles in DNA damage repair, transcriptional regulation, and cell death signaling.^[^ ^8^ ^]^ PARP1, the most predominant member of the PARP family, carries out more than 90% of the functions of the PARP family.^[^ ^9^ ^]^ To date, numerous PARP‐1 inhibitors have been developed, with six receiving approval for anticancer therapy.^[^ ^10^ ^]^ PARP1 inhibitors have been recognized as pivotal in addressing gemcitabine‐resistant pancreatic cancer.^[^ ^11^ ^]^ The discovery of novel PARP1 inhibitors may uncover new therapeutic strategies for tumors resistant to existing PARP1 inhibitors.^[^ ^12^ ^]^

Artemisinins and their derivatives, initially discovered for their anti‐malarial effects,^[^ ^13^ ^]^ are widely reported with a wide range of pharmacological activities, such as anti‐tumor,^[^ ^14^ ^]^ metabolic regulation,^[^ ^15^ ^]^ anti‐cardiac fibrosis,^[^ ^16^ ^]^ and treatment of polycystic ovary syndrome.^[^ ^17^ ^]^ Extensive preclinical evidence supports the anticancer properties of artemisinin derivatives, which exert their effects through various molecular mechanisms, including the induction of reactive oxygen species, inhibition of cell cycle progression, and initiation of apoptosis and ferroptosis in cancer cells.^[^ ^18^ ^]^ To elucidate their pharmacological mechanisms, our research has recently focused on the structural modification of artemisinin analogs and the identification of derivatives with superior physicochemical properties and therapeutic efficacy.^[^ ^19^ ^]^ Although artemisinin and its derivatives have the potential to be developed into novel antitumor drugs, the precise mechanisms of action remain to be fully elucidated and the specific cytotoxicity of artemisinin against tumor cells is not completely understood.

**Scheme 1:**
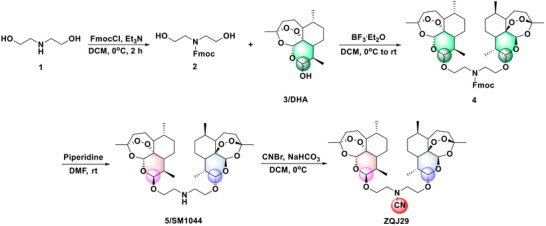
Synthesis procedures of ZQJ29.

ZQJ29 (**Scheme** **1**), a cyano‐substituted artemisinin dimer derivative, was synthesized by structural modification of artemisinin. Its evaluation for antitumor activity demonstrated a specific efficacy against pancreatic cancer. Subsequently, we proceeded to investigate its mechanism of action through proteomic analysis and functional assays, the pharmacological mechanisms underlying ZQJ29's efficacy against pancreatic cancer was elucidated. Our results showed that ZQJ29 inhibits PARP1 activity, thereby triggering ferroptosis in pancreatic cancer cells, and exhibited significant anti‐pancreatic cancer activity both in vitro and in vivo.

## 2 Results

### 2.1 Synthesis and Antitumor Activity Evaluation of Novel Artemisinin Derivatives

Extensive pharmacological investigations have established that artemisinin‐based dimers demonstrate significantly enhanced anticancer efficacy relative to their monomeric forms.^[^ ^20^ ^]^ Our research focuses on the structural optimization strategies for artemisinin derivatives based on our previous structure‐activity relationship studies.^[^ ^21^ ^]^ SM1044, a water‐soluble dimer incorporating aliphatic amine linkers, shows notable cytotoxicity against multiple cancer cell lines. To further enhance its efficacy, we implemented a rational design modification by introducing an electron‐withdrawing cyano group at the amine position of SM1044 to yield the novel artemisinin derivative ZQJ29 (Scheme 1) as a promising candidate with remarkable antitumor activity.

The cyano group, characterized by a highly polar carbon‐nitrogen triple bond and small steric bulk, can penetrate deeply into target proteins to form hydrogen bond interactions with key amino acid residues at the active site.^[^ ^22^ ^]^ Hydrogen‐bonding interactions enhance drug–target binding affinity, thereby increasing pharmacological activity and target selectivity.^[^ ^23^ ^]^ Additionally, the compact polar nature of the cyano group can enhance the aqueous solubility of the compound compared with SM1044. The structure of ZQJ29 was validated using ^1^H NMR, ^13^C NMR, and high‐resolution electrospray ionization mass spectrometry (ESI‐MS) (Figures S1–S3 and Table S1, Supporting Information). The purity of ZQJ29 was as high as more than 99%, as identified by HPLC (Figure S4, Supporting Information).

The cellular toxicity of the ZQJ29 compound was tested by the CCK‐8 assay on 8 cancer cell lines (PANC‐1, KP4, A549, H1975, JHH7, MCF‐7, HCT116, and HeLa) and 4 normal cell lines (HEK‐293T, LX2, THLE‐2, and HPDE6‐C7). Half‐maximal inhibitory concentrations IC_50_ values were shown in Table S2 (Supporting Information). ZQJ29 has demonstrated potent nanomolar‐range cytotoxicity against pancreatic cancer cell lines (PANC‐1: IC_50_ = 0.120 ± 0.030 µm, KP4: IC_50_ = 0.885 ± 0.106 µmM) and superior cytotoxic effects against pancreatic cancer cell lines compared to oxaliplatin (OXA) and relatively lower cytotoxicity toward normal cell lines, as outlined in Table S2 (Supporting Information). Notably, ZQJ29 displayed ≈100‐fold increased cytotoxicity against PANC‐1 cells and 10‐fold enhanced effect against KP4 cells when compared to the cytotoxicity of OXA and dihydroartemisinin (DHA), respectively. ZQJ29 showed significant selectivity for pancreatic cancer cell lines. PANC‐1 and KP4 were applied for subsequent study based on the cytotoxicity profile of ZQJ29.

### 2.2 Discovery of ZQJ29 as a Potent Inhibitor of Pancreatic Cancer In Vitro and In Vivo

**Figure 1:**
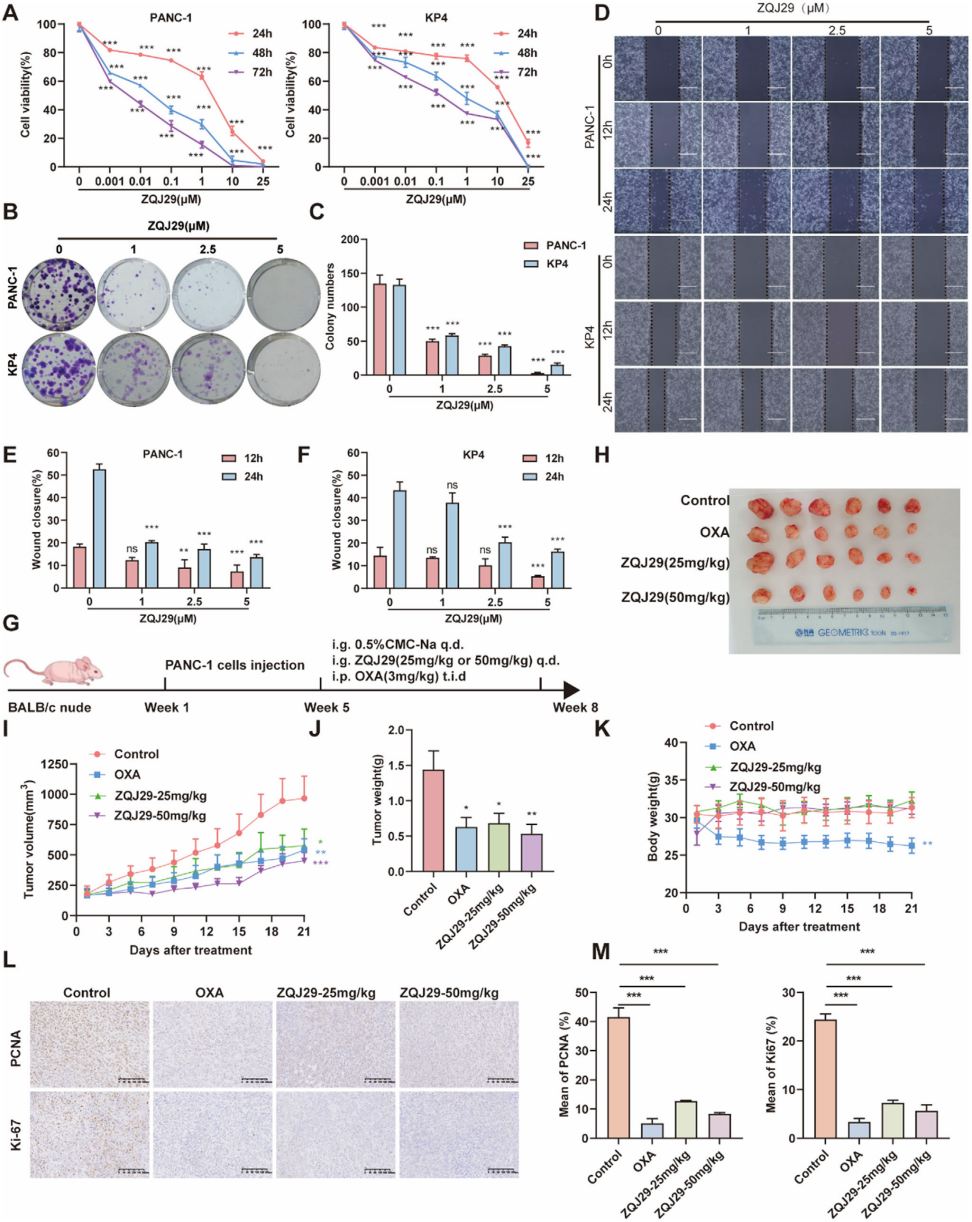
ZQJ29 inhibited the growth of pancreatic cancer cells in vitro and in vivo. A) PANC‐1 and KP4 cells were treated with increasing concentrations of ZQJ29, and CCK8 assay was performed after 24, 48, and 72 h. The bars indicate mean ± SD (*n* = 3). B,C) PANC‐1 and KP4 cells were seeded in 6‐well plates. After 12 h, cells were treated with indicated concentrations of ZQJ29. On the 10th day, the number of colonies was counted. The bars indicate mean ± SD (*n* = 3). D–F) PANC‐1 and KP4 cells in 6‐well plates were scratched to create a wound and starved in a serum‐free medium overnight followed by exposure to different concentrations of ZQJ29. Images were taken after 12 and 24 h of incubation at 37 °C. The bars indicate mean ± SD (*n* = 3). G) Scheme representing the experimental procedure. *n* = 6 mice per group. (i.g, intragastric; i.p, peritoneal injection; q.d, quaque die; t.i.d, ter in die) H) Illustrative depiction of solid tumors representative of different treatment groups in BALB/c nude mice. I) Tumor growth curves illustrated the progression in BALB/c nude mice across different treatment groups. The bars indicate mean ± SD (*n* = 6). J) The weights of the tumors in different groups. K) Body weight trends of the mice subjected to various treatment regimens. The bars indicate mean ± SD (*n* = 6). L) IHC staining of PCNA and Ki67 proteins in tumor tissues. Scale bar: 200 µm. M) Statistical analysis of IHC. ^*^ *p *< 0.05; ^**^ *p* < 0.01; ^***^ *p* < 0.001; ns, not significant.

PANC‐1 and KP4 cell lines were used to evaluate the antiproliferative effects of ZQJ29 on pancreatic cancer. As shown in **Figure** **1**A, ZQJ29 effectively inhibited the cellular viability in a dose‐dependent and time‐dependent manner. Colony formation assays, which serve as a reliable in vitro model for simulating the pathological processes of cancer progression observed in vivo, demonstrated that ZQJ29 significantly inhibited the colony formation of PANC‐1 and KP4 cells in a concentration‐dependent manner (Figure 1B,C). Additionally, cell migration assays showed that the migration abilities of PANC‐1 and KP4 cells were impaired after incubation with ZQJ29 (Figure 1D–F). These results indicated that ZQJ29 possessed robust antiproliferative activity against cancer cells in vitro.

Male BALB/c nude mice were subcutaneously implanted with PANC‐1 cells to evaluate the in vivo antitumor activity of ZQJ29. Mice‐bearing PANC‐1 tumors were treated with 0.5% CMC‐Na, ZQJ29, or OXA (Figure 1G). Tumor growth was markedly increased in control mice, while it was effectively inhibited in both ZQJ29 and OXA‐treated groups. In addition, compared to the control group, administration of ZQJ29 at 25 or 50 mg kg^−1^ significantly reduced tumor size and weight (Figure 1H–J). During the whole treatment period, the body weight of ZQJ29‐treated mice remained stable, whereas that of OXA‐treated mice decreased significantly (Figure 1K). The post‐experiment levels of ALT and AST showed no significant alterations in the mice treated with ZQJ29 at 50 mg kg^−1^, however, it was significantly elevated in the OXA‐treated group (Figure S5, Supporting Information). Furthermore, H&E staining indicated no significant major organ damage in ZQJ29‐treated mice, in contrast to OXA‐treated mice (Figure S6, Supporting Information). Collectively, these observations highlighted that the administration of ZQJ29 did not induce any widespread toxic effects on the mice. The expression of proliferating cell nuclear antigen (PCNA) and nuclear antigen (Ki‐67) was examined to elucidate the mechanisms of ZQJ29 on cancer development. IHC staining demonstrated significantly reduced expression of Ki67 and PCNA following ZQJ29 and OXA treatment, suggesting that ZQJ29 effectively suppressed pancreatic cancer progression (Figure 1L–M). Additionally, the pharmacokinetic parameters indicated that ZQJ29 had a t_1/2_ of 1.56 h and an average residence time (MRT_0‐inf_) of 2.74 h (Table S3, Supporting Information), which was significantly superior to those of DHA (t_1/2_ = 0.5 h).^[^ ^24^ ^]^

In short, these results indicated that ZQJ29 was superior to the marketed drug OXA in anti‐ pancreatic cancer both in vivo and in vitro.

### 2.3 Ferroptosis was Identified as a Primary Determinant of ZQJ29‐Induced Cell Death in Pancreatic Cancer Cells

**Figure 2:**
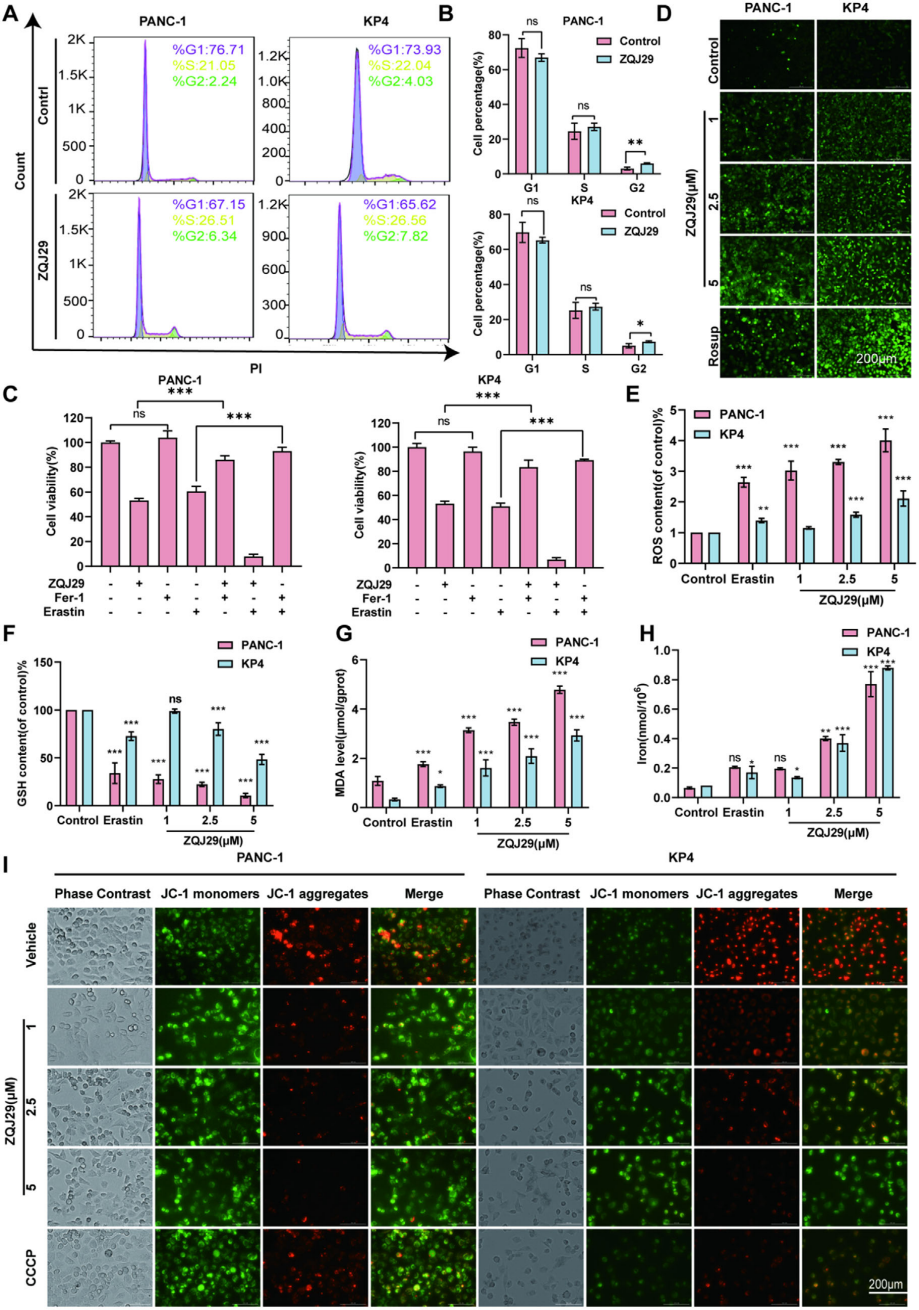
ZQJ29 induced cell cycle arrest and ferroptosis in PANC‐1 and KP4 cells. A,B) PANC‐1 and KP4 cells were left untreated or treated with ZQJ29 at the indicated doses for 24 h, and cell cycles were labeled with PI and analyzed by flow cytometry. The quantitative data from (A) is shown in (B). C) Cell viability of PANC‐1 and KP4 cells after treatment of the combination of ZQJ29 (0.5 µm) with ferroptosis inhibitor Ferrostatin‐1 (10 µm), and ferroptosis activator Erastin (10 µm) for 24 h. D) The ROS (green fluorescence) in PANC‐1 and KP4 cells after incubation with different concentrations of ZQJ29 for 24 h. Scale bar: 200 µm. E–H) The ROS, intracellular iron, MDA, and GSH levels after different concentrations of ZQJ29 were treated for 24 h in PANC‐1 and KP4 cells. I) The JC‐1 (green fluorescence) in PANC‐1 and KP4 cells after incubation with different concentrations of ZQJ29 for 24 h. Scale bar: 200 µm. The bars indicate mean ± SD (*n* = 3). ^*^p < 0.05; ^**^p < 0.01; ^***^p < 0.001; ns, not significant.

Cell cycle arrest represents one of the major approaches by which most clinical chemotherapies kill cancer cells.^[^ ^25^ ^]^ Flow cytometric analyses were thereby conducted to examine whether ZQJ29 induced the cycle arrest in PANC‐1 and KP4 cells. Next, the cell cycle distributions of PANC‐1 and KP4 cells after exposure to ZQJ29 were examined. ZQJ29 induced a remarkable G_2_/M phase arrest in the two cell lines (**Figure** **2**A,B). To observe the mechanisms of cell death induced by ZQJ29, several inhibitors of cell death pathways were applied in the co‐culture systems. Co‐treatment with Z‐VAD‐FMK (an apoptosis inhibitor), 3‐MA (an autophagy inhibitor), or Nec‐1 (a necroptosis inhibitor), did not reverse ZQJ29‐ induced cell death in both PANC‐1 and KP4 cells (Figure S7, Supporting Information), suggesting that apoptosis, autophagy, and necroptosis were not the primary events triggered by ZQJ29. Interestingly, co‐treatment with Fer‐1 (a ferroptosis inhibitor) reversed ZQJ29‐induced cell death, an effect comparable to that of Erastin (a ferroptosis activator) on PANC‐1 and KP4 cells (Figure 2C). This finding implies that ZQJ29 is likely to exert its antitumor effects by inducing ferroptosis. With further research, accumulating evidence has supported the significance of ferroptosis in cancer cell death.^[^ ^26^ ^]^ To determine the role of ferroptosis in ZQJ29‐induced cell death, we analyzed the intracellular levels of lipid reactive oxygen species (ROS), iron, glutathione (GSH), and malondialdehyde (MDA), which are key parameters for quantifying ferroptosis^[^ ^27^ ^]^ in PANC‐1 and KP4 cells treated with ZQJ29. The results demonstrated increased ROS release, iron accumulation, MDA production, and GSH depletion following 24 h of ZQJ29 treatment (Figure 2D–H). Furthermore, changes in mitochondrial membrane potential (MMP), a critical event in ferroptosis, were visualized using JC‐1 staining. High MMP typically yields red fluorescence, which shifts to green fluorescence when MMP is reduced.^[^ ^28^ ^]^ Our data revealed a decrease in MMP in PANC‐1 and KP4 cells (Figure 2I) following the treatment with ZQJ29 for 24 h, as indicated by increased green fluorescence. This result indicated that ZQJ29 can reduce MMP and impair mitochondrial function. Taken together, these findings strongly suggested that ZQJ29‐induced cell death in pancreatic cancer cells was achieved primarily by induction of ferroptosis.

### 2.4 Proteomic Study of ZQJ29 in Pancreatic Cancer Cells

**Figure 3:**
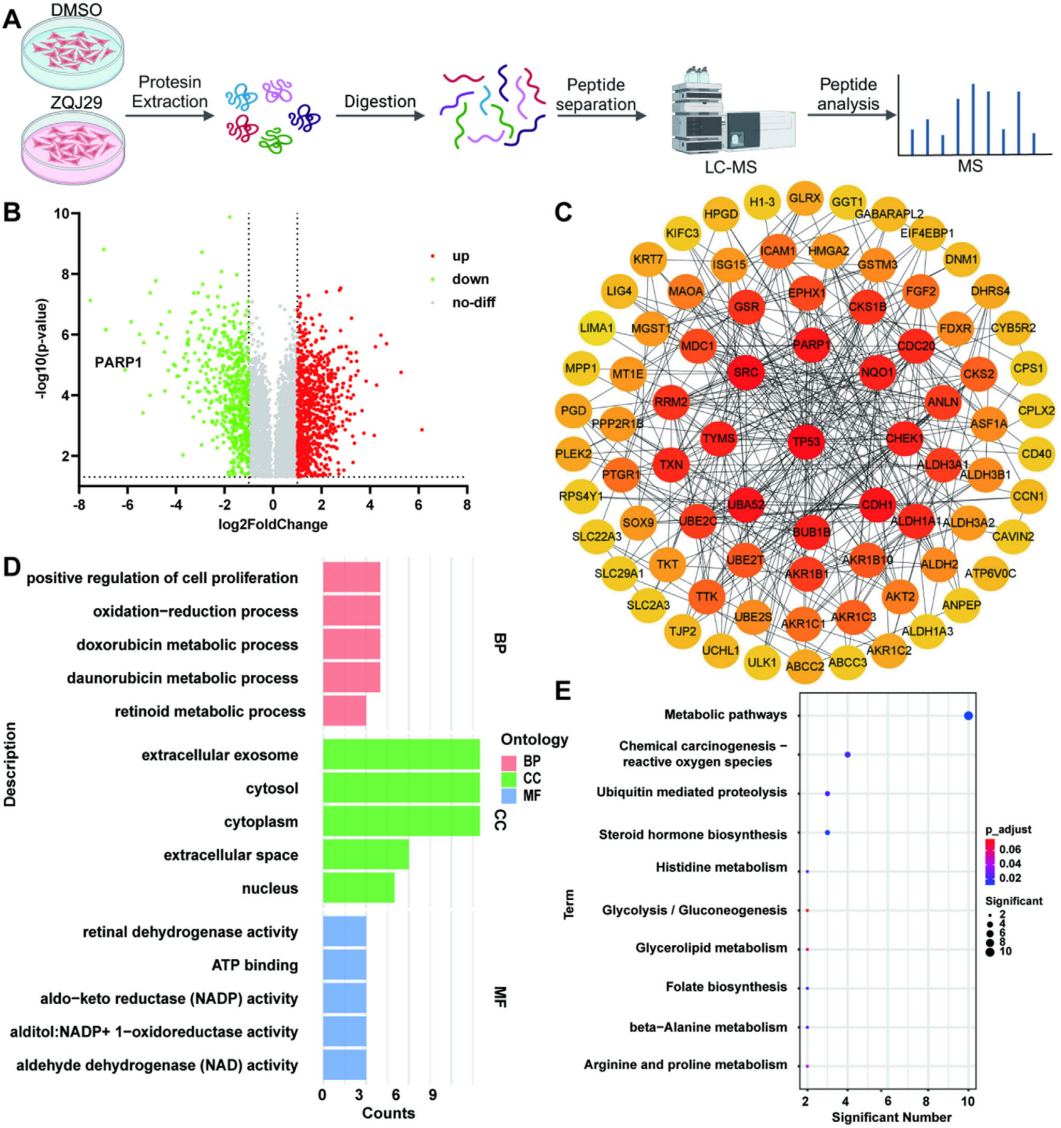
Proteomic study of ZQJ29 in pancreatic cancer cells. A) Brief description of the process for proteomics. B) The volcano plot exhibited the differentially expressed genes from proteomics data after treatment with ZQJ29 or DMSO for 24 h. The log2 fold change (FC) was plotted versus the –log10 of the p‐value. The values indicate the expression of the proteins, red dots (up): hit with *p *< 0.05 and mean log2FC > 1; green dots (down): hit with *p *< 0.05 and mean log2FC < ‐1. C) PPI analysis among the top 100 significantly different proteins. (D‐E) KEGG enrichment and GO enrichment analyses of differentially expressed genes.

To explore the targets and mechanisms of ZQJ29 in the treatment of pancreatic cancer, we conducted the proteomic study using pancreatic cancer cell line PANC‐1 (with or without ZQJ29 treatment). **Figure** **3**A showed a brief flowchart of proteomics. A total of 5443 proteins were identified, with 1386 differentially expressed proteins (DEPs) discerned between the two groups using a fold change criterion of FC > 2 or FC < 0.5 and *P* < 0.05 as the screening condition. Among these DEPs, 555 proteins were downregulated, and 831 proteins were upregulated. Notably, PARP1 was prominently down‐regulated (Figure 3B). PARP1 is known to be overexpressed in human tumors^[^ ^29^ ^]^ and its protein function is regulated by post‐translational modification with ADP ribose units using NAD^+^ as a substrate.^[^ ^30^ ^]^ Subsequently, the top 100 DEPs were subjected to protein‐protein interactions (PPI) analysis using the STRING database and the interactions were visualized by constructing a PPI network diagram with Cytoscape 3.9.1 software. The gene target of TP53 in the top 100 DEPs was involved in the most biological functions (Figure 3C). TP53, the most commonly mutated tumor suppressor gene, is one of the prominent keys linking ferroptosis to tumor suppression.^[^ ^31^ ^]^

### 2.5 ZQJ29 Inhibits PARP1 Activity Through Direct Binding

**Figure 4:**
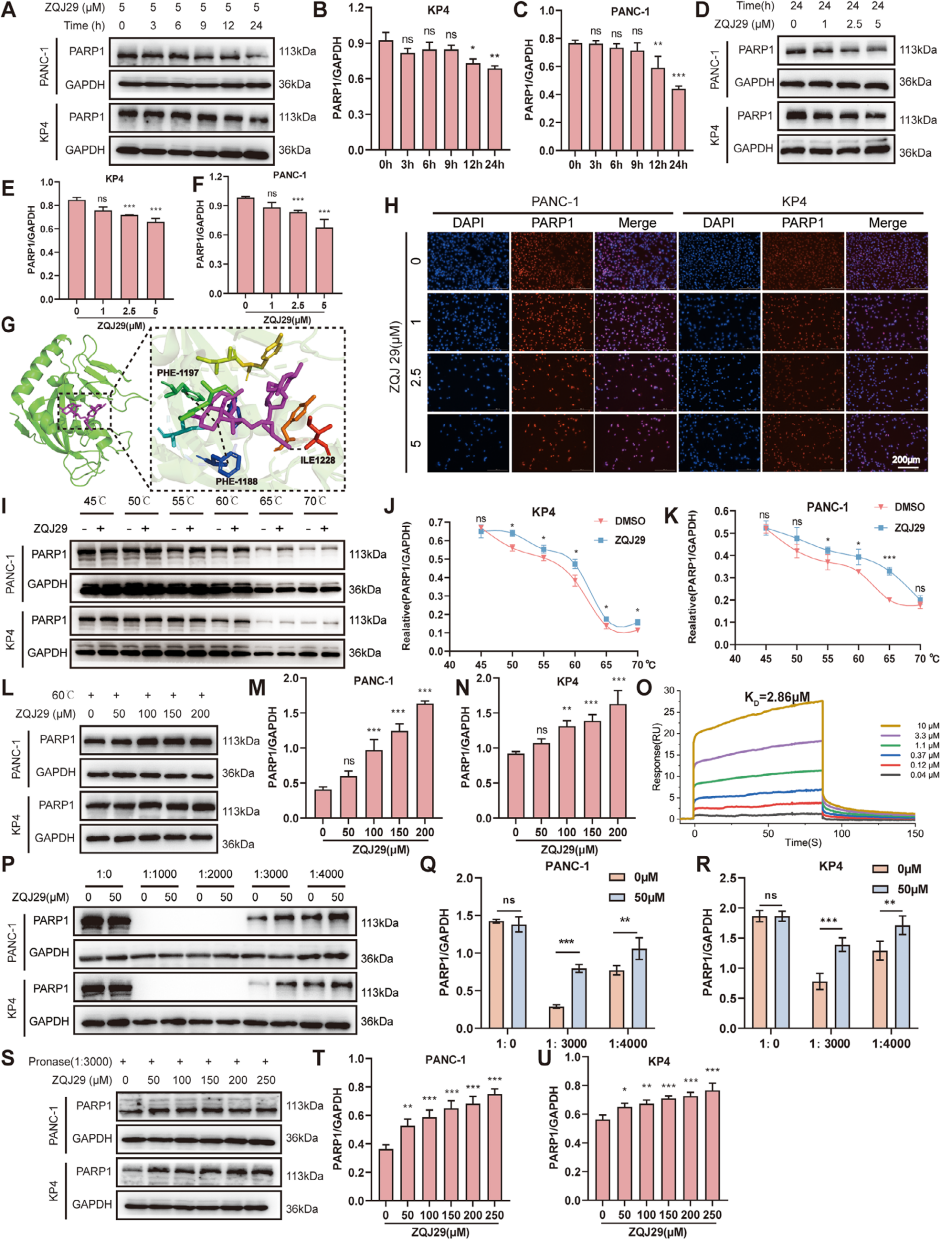
ZQJ29 inhibits PARP1 activity through direct binding with PARP1. A) PANC‐1 and KP4 cells were treated with varying concentrations of ZQJ29 for 24 h. B,C) Statistical analysis of Figure 4A. D) PANC‐1 and KP4 cells were treated with 5 µM of ZQJ29 for specified periods. E,F) Statistical analysis of Figure 4D. G) Molecular docking model illustrating the binding interaction between ZQJ29 and PARP1. H) Immunofluorescence staining of PARP1 (red) and nuclear DAPI staining (blue) in PANC‐1 and KP4 cells after 24 h treatment with ZQJ29 (0, 1, 2.5, and 5 µm). Scale bar: 200 µm. I) Thermal stability analysis of PARP1‐ZQJ29 interaction using CETSA across a temperature gradient (45‐70 °C). (J–K) Statistical analysis of Figure 4I. L) PARP1 stability at 60 °C under treatment with different ZQJ29 concentrations. M,N) Statistical analysis of Figure 4L. O) SPR assay. P) Stability of PARP1 treated with varying pronase/protein ratios. Q,R) Statistical analysis of Figure 4P. S) PARP1 stability under different ZQJ29 concentrations (1:3000). T,U) Statistical analysis of Figure 4S. The data was shown as mean value ± SD. ^*^ *p* < 0.05; ^**^ *p* < 0.01; ^***^ *p* < 0.001; ns, not significant.

To verify the previous proteomics results, PANC‐1 and KP4 pancreatic cancer cells were treated with ZQJ29. The results showed that PARP1 expression in PANC‐1 and KP4 cells were significantly reduced in time‐dependent and concentration‐dependent manners after ZQJ29 treatment (**Figure** **4**A–F). Next, immunofluorescence assays were conducted to investigate whether ZQJ29 regulates nuclear PARP1 expression, and the results indicated that ZQJ29 remarkably reduced nucleoplasmic PARP1 levels in PANC‐1 and KP4 cells (Figure 4H). To further identify the amino acid residues of PARP1 that serve as binding sites for ZQJ29, the binding mode was explored using Autodock Vina software.^[^ ^32^ ^]^ As illustrated in Figure 4G and Figure S8 (Supporting Information), the hydrogen bond was observed between the ZQJ29's cyano group and amino acid residue ILE1128. To identify whether ZQJ29 was directly bound to the PARP1 protein, a cellular thermal shift assay (CETSA) was performed. As shown in Figure 4I–K, PARP1 accumulation was significantly increased from 45  to 70 °C with ZQJ29 treatment, suggesting a direct ZQJ29 interaction with PARP1 on thermal stability. To further validate the CETSA results, cell lysates were treated with varying concentrations of ZQJ29 at 60 °C and the results showed that PARP1 stability was enhanced with increasing ZQJ29 concentrations (Figure 4L–N). Surface plasmon resonance (SPR) analysis showed that ZQJ29 could bind to the PARP1 protein with a higher affinity (K_D_ = 2.86 µm, Figure 4O). Consistent with CETSA findings, as the enzyme concentration decreases, PARP1 accumulation also decreases (Figure 4P–R). PARP1 accumulation increased with higher concentrations of ZQJ29 when the enzyme‐lysate ratio was 1:3000 (Figure 4S–U). Collectively, these results suggested that ZQJ29 inhibits PARP1 activity through direct binding.

### 2.6 ZQJ29‐Induced Ferroptosis is Dependent on PARP1

**Figure 5:**
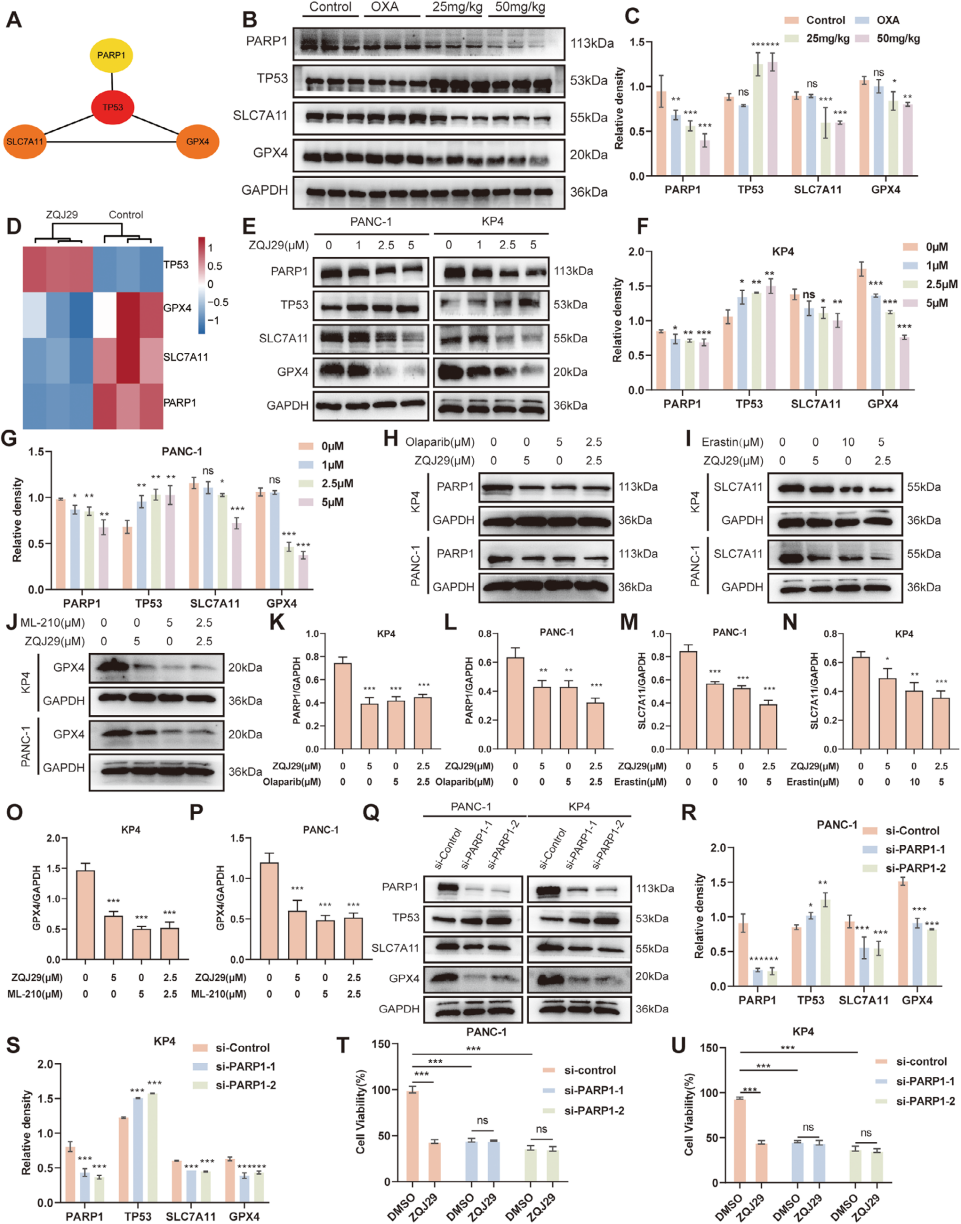
ZQJ29‐induced ferroptosis is PARP1‐dependent. A) PPI network analysis. B) Protein expression in mouse tumor tissues. C) Statistical analysis of Figure 5B. D) Heatmap of protein expression from proteomic data. E) Protein expression in PANC‐1 and KP4 cells treated with varying concentrations of ZQJ29. F,G) Statistical analysis of Figure 5E. H–J) Protein expression in PANC‐1 and KP4 cells treated with ZQJ29 alone or in combination with PARP1 inhibitor (Olaparib), SLC7A11 inhibitor (Erastin), or GPX4 inhibitor (ML‐210). K–P) Statistical analysis of Figure 5H–J. Q) Expression of PARP1, TP53, SLC7A11, and GPX4 in PANC‐1 and KP4 cells transfected with different PARP1 siRNAs. R,S) Statistical analysis of Figure Q. T,U) Cell viability assessed by CCK‐8 assay in PANC‐1 and KP4 cells transfected with control‐siRNA or PARP1‐siRNA, followed by treatment with ZQJ29 (1 µm) for 24 h. The data was shown as mean value ± SD. ^*^p < 0.05; ^**^p < 0.01; ^***^p < 0.001; ns, not significant.

Based on the above analyses, we concluded that ZQJ29 promotes ferroptosis in pancreatic cancer cells. Subsequent proteomic analyses confirmed PARP1 as a potential target, and ZQJ29 was proved to inhibit the activity of PARP1 by direct binding. Ferroptosis is characterized by an iron‐dependent mode of programmed cell death, primarily regulated by the excessive accumulation of lipid peroxides due to the downregulation of the SLC7A11/GPX4 axis.^[^ ^33^ ^]^ Inhibition of PARP1 promotes TP53 expression, while TP53 reduces SLC7A11 expression by directly binding to the SLC7A11 promoter or interacting with ubiquitin‐specific protease 7, and SLC7A11 downregulates GPX4 protein expression in ferroptosis lipid peroxidation.^[^ ^34^ ^]^ Subsequently, we subjected PARP1, TP53, SLC7A11, and GPX4 to PPI analysis using the STRING database and visualized by constructing a PPI network diagram using Cytoscape 3.9.1 software. The STRING network diagram revealed that PARP1 interacted with TP53, which subsequently interacted with SLC7A11 and GPX4 (**Figure** **5**A). The tumor tissue and proteomic analyses revealed that following ZQJ29 intervention, PARP1, SLC7A11, and GPX4 were downregulated, while TP53 was upregulated (Figure 5B–D). To elucidate the mechanism by which PARP1 promotes ferroptosis, we examined the expression of the above four proteins in PANC‐1 and KP4 cell lines. The results showed that the expression of PARP1, SLC7A11, and GPX4 was downregulated, while TP53 was upregulated by ZQJ29 in a dose‐dependently manner (Figure 5E–G). Consistent with the effects of PARP1 inhibitor (Olaparib, 5 µm), SLC7A11 inhibitor (Erastin, 10 µm) and GPX4 inhibitor (ML‐210, 5 µm), ZQJ29 significantly inhibited the expression of PARP1, SLC7A11, and GPX4. Furthermore, synergistic effects were observed when these inhibitors were used in combination with ZQJ29 (Figure 5H–P). To assess the effect of PARP1 on the expression of the three proteins, we silenced PARP1 using specific small interfering RNA (siRNA). PARP1 knockdown resulted in a significant decrease in the SLC7A11 and GPX4 protein levels, while inducing the overexpression of TP53 in both PANC‐1 and KP4 cells (Figure 5Q–S). Notably, the proliferation inhibition of PANC‐1 and KP4 cells induced by ZQJ29 was markedly reversed upon PARP1 knockdown through PARP1‐siRNA transfection (Figure 5T–U). Overall, ZQJ29 acts as a PARP1 inhibitor that downregulates the expression of SLC7A11 and GPX4 in a TP53‐dependent manner, thereby promoting ferroptosis in pancreatic cancer cells.

## 3 Discussion

Numerous studies have demonstrated that artemisinin and its derivatives exhibit significant cytotoxicity against various cancer cells, including pancreatic cancer cells.^[^ ^35^ ^]^ However, the precise pharmacological targets of artemisinin remain unclear. Research on the structural modification of artemisinin has shown that the dimer of artemisinin has a markedly stronger pharmacological effect than the monomer, with several orders of magnitude higher activity in inhibiting tumor growth,^[^ ^36^ ^]^ artemisinin and its derivatives have emerged as promising candidate drugs for cancer therapy. Herein, a cyano‐substituted artemisinin dimer derivative, ZQJ29 was synthesized through structural modification of artemisinin. ZQJ29 demonstrates unique therapeutic advantages over conventional artemisinin derivatives through three synergistic innovations. Structurally, N‐cyano dimer conformation of ZQJ29 enhances its targeting properties, as shown by the molecular docking results, which indicate that the cyano portion forms hydrogen bonds with the target protein. Pharmacologically, ZQJ29 exhibited a 10‐fold potency of antitumor over DHA. Dimeric structure of ZQJ29 facilitates dual targeting of PARP1 and the ferroptosis pathway, and amplifies ROS generation to synergistically drive ferroptosis in cancer cells. Furthermore, preliminary pharmacokinetic studies (Table S4, Supporting Information) revealed that ZQJ29 (t_1/2_ = 1.6 h) possesses a prolonged plasma half‐life compared to DHA (t_1/2_ = 0.5 h), indicating an improvement in bioavailability and feasibility of drug administration. With mechanistic and ADME optimizations through strategic structural modification, ZQJ29 emerges as a potential candidate that bridges the therapeutic advantage of artemisinin with the precision‐targeting requirements in modern oncology. PARP1 was first identified as the target of ZQJ29 in the treatment of pancreatic cancer, and ZQJ29 targets PARP1 to activate TP53, thereby inhibiting the SLC7A11/GPX4 signaling pathway and promoting ferroptosis in pancreatic cancer cells. There was a complex and multifaceted relationship between ferroptosis and tumor development. Ferroptosis is an iron‐dependent, non‐apoptotic form of programmed cell death, characterized by elevated intracellular iron levels, and ferroptosis induces the accumulation of lipid peroxides to lethal levels.^[^ ^37^ ^]^ Consistent with our findings, ZQJ29 inhibits the proliferation of pancreatic cancer cells, accompanied by the release of ROS, accumulation of iron, and depletion of GSH, leading to MDA production and reduction of MMP. This indicates that cell ferroptosis induced by ZQJ29 occurs through the lipid peroxidation pathway.

PARP1 is a DNA repair enzyme involved in repairing damaged DNA in cells. Previous studies have reported that the expression of PARP1 in pancreatic cancer tissues is significantly higher than that in adjacent non‐cancerous tissues.^[^ ^38^ ^]^ In this study, PARP1 was identified as a potential target in pancreatic cancer by proteomics analysis. Subsequently, PARP1 was proven as a direct binding target of ZQJ29 by molecular docking, CETSA, and DARTS. More importantly, ZQJ29 was showed no toxic side effects and had comparable efficacy to the chemotherapeutic drug OXA in vivo.

Interestingly, we observed that ZQJ29‐induced cell death was reversed by the ferroptosis inhibitor ferrostatin‐1 (Fer‐1), suggesting that ZQJ29 triggers ferroptosis through PARP1. It has been reported that PARP inhibitors can promote the activation of the TP53 signaling pathway,^[^ ^39^ ^]^ while tumor suppressor factors can inhibit cancer progression by regulating the expression of ferroptosis‐related gene or proteins. For example, TP53 can inhibit the expression of SLC7A11, thereby enhancing the sensitivity of tumor cells to ferroptosis. Our study showed that TP53 in pancreatic cancer cells significantly increased after treatment with ZQJ29, and the SLC7A11/GPX4 axis in ferroptosis was suppressed, this indicated a high correlation between PARP inhibition and ferroptosis. Subsequently, we conducted PARP1 knock‐down experiment in PANC‐1 and KP4 cell lines, observing characteristics of ferroptosis, including the upregulation of TP53 and downregulation of SLC7A11/GPX4 expression. These results indicated that downregulation of PARP1 promotes cell ferroptosis. Moreover, in the gene‐knockdown PANC‐1 and KP4 pancreatic cancer cells, ZQJ29 treatment no longer inhibited their activity. This indicated that ZQJ29 induced ferroptosis in pancreatic cancer cells through a PARP1‐dependent pathway.

**Figure 6:**
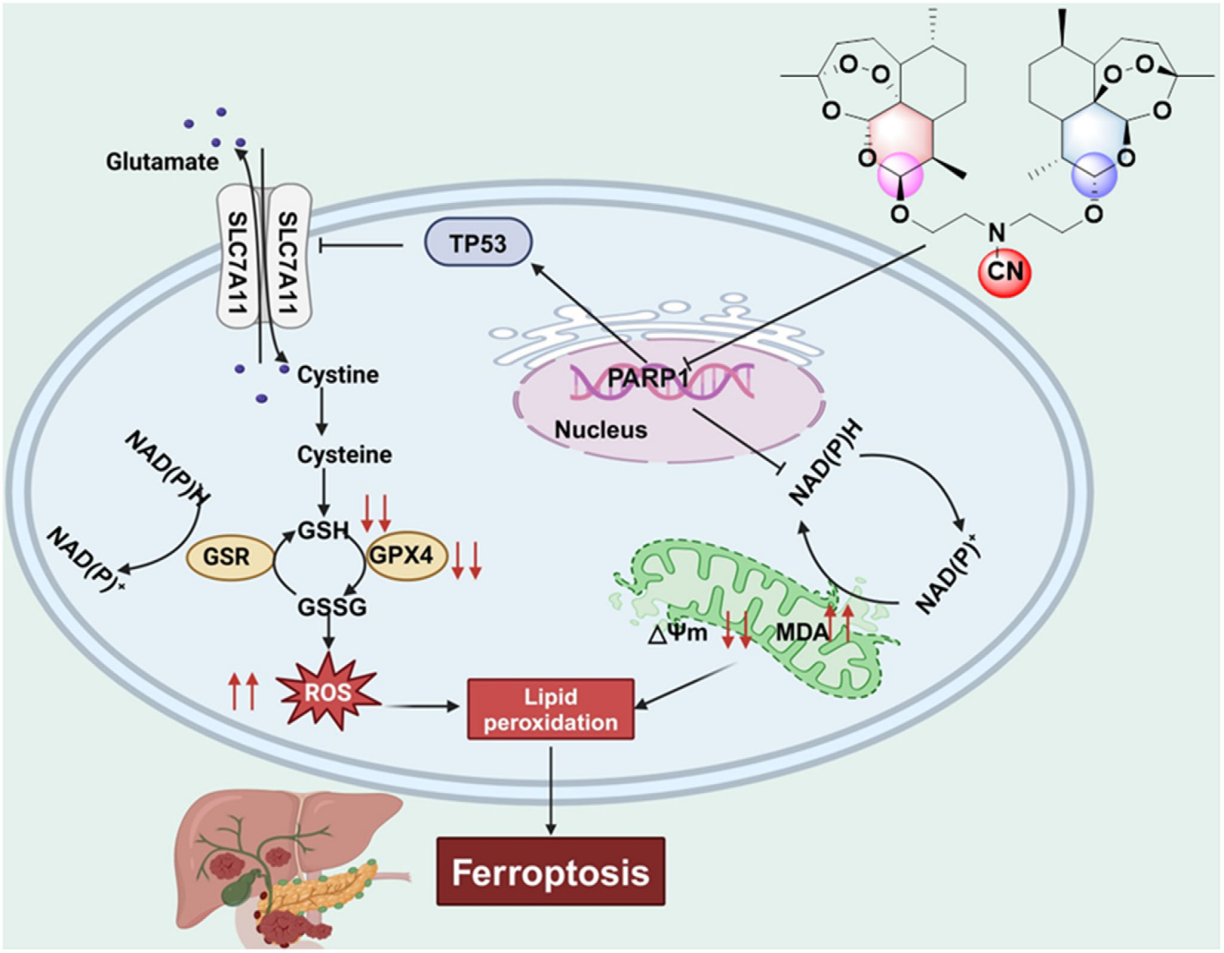
The schematic illustration for ZQJ29 targeted PARP1 to activate ferroptosis for anti‐pancreatic cancer.

The clinical recalcitrance of pancreatic adenocarcinoma, stemming from its genomic heterogeneity and resistance to monotherapy, innovative multi‐targeted therapeutic strategies was urgent. This study demonstrates that ZQJ29, a rationally designed novel artemisinin dimer derivative, achieves synergistic antitumor efficacy through concurrent targeting of PARP1 and potentiation of ferroptosis (mechanistic details illustrated in **Figure** **6**). This multi‐targeted strategy could effectively resolve critical clinical challenges including drug resistance and limitations of monotherapy. Notably, ZQJ29 exhibits comparable tumor‐suppressive efficacy to the first‐line clinical chemotherapeutic agent OXA, while exhibiting superior safety profiles characterized by the absence of significant organ toxicity and preserved hepatic function (ALT/AST levels within normal ranges). Collectively, these findings underscore the potential of ZQJ29 as a viable therapeutic candidate. The PARP1‐targeted mechanism for inducing ferroptosis demonstrates promising capabilities to address critical clinical challenges in pancreatic cancer therapy.

## 4 Conclusion

In this study, ZQJ29, a novel artemisinin derivative with significant potential as an anti‐pancreatic cancer agent and a promising PARP1 inhibitor with a peroxide bridge structure, was synthesized. Its anticancer efficacy, particularly in inducing ferroptosis, was verified through both in vitro experiments on PANC‐1 and KP4 cell lines and in vivo studies using animal models. The mechanistic insights into the anticancer properties of ZQJ29 were delineated as follows: inhibition of PARP1 led to the generation of NAD(P)^+^, which decreased MMP and increased MDA levels, subsequently promoting ferroptosis. Additionally, inhibition of PARP1 promoted TP53 expression and inhibited the SLC7A11/GPX4 pathway, further facilitating ferroptosis in pancreatic cancer cells. In summary, ZQJ29 is a molecular‐targeted anticancer candidate with significant lower toxicity compared to the clinically approved therapeutic OXA in vivo. We have discovered a novel artemisinin derivative with PARP1‐targeted activity for the first time. This study provides compelling evidence that ferroptosis can be induced through specific targeting of PARP1, offering new insights and innovative therapeutic strategies for addressing pancreatic cancer.

## 5 Experimental Section

### Synthesis and Characterization

Commercially available high‐purity diethanolamine was used as the starting material, and its amino group was protected by fluorenylmethoxycarbonyl (F‐moc) at low temperature to obtain compound 2. This compound was then reacted with DHA (*α* and *β*) in a mixed conformation to yield compound 4. The F‐moc protecting group was subsequently removed to produce SM1044. Next, SM1044 (120 mg, 0.19 mmol) was dissolved in DCM (7 mL) and stirred under N_2_ protection of at 0 °C. Aqueous sodium bicarbonate (63 mg, 0.75 mmol, 1 mL) was added dropwise, followed by cyanogen bromide (24 mg, 0.22 mmol) dissolved in DCM (1 mL), which was also added dropwise. The reaction was allowed to proceed overnight for 12 h. At the end of the reaction, the solvent was evaporated under reduced pressure, yielding a small amount of white precipitate. Deionized water was then added (10 mL), and the mixture was extracted with DCM (10 mL × 3). The organic phases were combined, dried over anhydrous Na₂SO₄, filtered to remove the Na₂SO₄, and concentrated under vacuum. Finally, the crude product was purified by silica gel column chromatography (dichloromethane: methanol = 80: 1), and a white solid compound ZQJ29 (79 mg, 63.4%) was obtained. ^1^H NMR (400 MHz, CDCl_3_) δ 5.43 (s, 2H), 4.82 (d, *J* = 3.6, 2H), 4.05‐4.00 (m, 2H), 3.61‐3.56 (m, 2H), 3.33‐3.22 (m, 4H), 2.69‐2.62 (m, 2H), 2.40‐2.32 (m, 2H), 2.06‐2.00 (m, 2H), 1.91‐1.85 (m, 2H), 1.80‐1.75 (m, 4H), 1.67‐1.62 (m, 4H), 1.53‐1.47 (m, 3H), 1.42 (s, 6H), 1.38‐1.35 (m, 1H), 1.27‐1.22 (m, 2H), 0.95‐0.93 (m, 14H). ^13^C NMR (100 MHz, CDCl_3_) *δ* ppm: 117.30, 104.33, 102.63, 88.16, 81.15, 65.73, 52.68, 51.75, 44.44, 37.50, 36.53, 34.72, 30.87, 26.26, 24.80, 24.54, 20.50, 13.15. HR‐MS (ESI): calcd for C_35_H_54_N_2_O_10_ ([M +H]^+^): 663.3854, found: 663.3854.

### Cell Viability Assay

Cell viability was evaluated using the Cell Counting Kit‐8 (Beyotime, Shanghai, China) assay. Cells were seeded in 96‐well plates at a density of 2×10^3^ cells to allow for attachment. After treatment with ZQJ29 (0.001, 0.01, 0.1, 10, and 25 µm) for 24, 48, and 72 h, 10 µL of CCK‐8 solution was added to each well and incubated for 1 h. The optical density values were then measured at 450 nm using a multifunctional enzyme‐linked immunosorbent assay reader (Tecan, Spark, Switzerland).

### Colony Formation Assay

The clonogenicity of pancreatic cancer cells was evaluated using a colony formation assay. PANC‐1 and KP4 cells were seeded into 6‐well plates at ≈200 cells per well in the conditional medium. The cells were treated with different concentrations of ZQJ29 (0, 1, 2.5, and 5 µm) for 24 h. After treatment, the drug‐containing medium was removed, and complete medium was added for an additional 24 h in a 37 °C incubator with 5% CO_2_. The plates were then washed with PBS and fixed with 4% paraformaldehyde. After staining with 0.5% crystal violet (Yeasen, Shanghai, China), the colonies were counted manually.

### Wound Healing Assay

PANC‐1 and KP4 cells were cultured in 6‐well plates until complete confluence was reached. A “scratch” or “wound” was made with a sterile 10 µL pipette tip. Following this, the cells were treated with varying concentrations of ZQJ29 (0, 1, 2.5, and 5 µm). After 12  and 24 h, migration activity were observed and captured using an inverted EVOS microscope (Thermo Fisher Scientific, USA). The number of migrated cells were then quantified.

### Animal Experiments

All animal experiments were approved by the Ethics Committee for Animal Experiments of Shanghai University of Traditional Chinese Medicine (Approval No: PZSHUTCM2302130002; PZSHUTCM2302260001).

### Pharmacokinetic Study

Male Sprague‐Dawley (SD) rats (200 ± 20 g) were purchased from Beijing Vital River Laboratory Animal Technology Co., Ltd. (Beijing, China). After a one‐week acclimatization period, the rats were injected intragastrically with ZQJ29 (50 mg kg^−1^). Blood samples were collected from the jugular veins after the injection of ZQJ29 (at 5, 10, 20, 40, 60, 120, 240, 360, 720, and 1440 min). The whole blood was centrifuged at 4000 × g for 10 min at 4 °C. Plasma was extracted with MeOH by vortexing for 10 min. After centrifugation for 12 min at 12 000 × g at 4 °C, the supernatant was analyzed using an HPLC‐MS/MS system. The concentration of ZQJ29 was quantitatively measured by the external standard method. Pharmacokinetic parameters were calculated using Drug and Statistic (DAS, version 2.0) software based on the compartmental method.

Five‐week‐old male BALB/c nude mice were purchased from Shanghai Jihui Laboratory Animal Breeding Co., Ltd. (Shanghai, China). After a one‐week acclimatization period, PANC‐1 cells were subcutaneously injected at a dose of 5 × 10^6^ cells per mouse. Four weeks post‐injection, once tumor volumes reached 50–100 mm^3^, the tumor‐bearing animals were randomly divided into four groups, each consisting of six animals. ZQJ29 was prepared in a 0.5% CMC‐Na solution, while OXA was dissolved in saline. The control group was treated with oral gavage of 0.5% CMC‐Na, while the positive control group was administered intraperitoneal injections of OXA at a dosage of 3 mg kg^−1^ every three days. The test groups were treated with oral gavage of ZQJ29 at dosages of 25  and 50 mg kg day^−1^. Mouse weights were monitored every other day. Tumor measurements were taken every other day using calipers, and the tumor volume was calculated using the following formula: ½ × length × width^2^. Drug administered continued for three weeks. After blood collection at the end of the administration, the mice were humanely euthanized, and their vital organs were promptly harvested, preserved, and embedded in paraffin for H&E staining.

### H&EStaining

Following the experiments, organs from mice, including hearts, livers, spleens, lungs, and kidneys were swiftly collected and fixed in 4% paraformaldehyde overnight. The tissues were then embedded in paraffin. Afterward, 4‐µm‐thick sections were produced and stained using H&E. Images of these sections were captured with a Leica DM4000b microscope (Germany).

### Blood Biochemical Test

Blood samples were collected and placed in a biochemical incubator for 30 min, followed by centrifugation at 6000 rpm for 10 min. The supernatant was used to measure the following indicators: ALT and AST (both from Jiancheng, Nanjing, China).

### Protein Extraction and Label‐Free Proteomics

PANC‐1 cells were inoculated into 6‐well plates at ≈2 × 10^5^ cells per well and grown for 24 h. After this period, the cells were treated with 5 µm ZQJ29 and an equal volume of solvent (DMSO) for an additional 24 h. Following treatment, the cells were removed from the incubator, and the medium was aspirated and discarded. The cells were washed twice with pre‐cooled PBS, which was then discarded. Next, 30 µL of RIPA lysis buffer containing PMSF (Yeasen, Shanghai, China) was added, and the cells were scraped off quickly using a cell scraper. The cell suspension was collected in 1.5 mL EP tubes and placed on ice. Cells were homogenized using ultrasound (3s × 15 times at 4s intervals), and the mixture was kept on ice for 10–15 min until the foam subsided. The centrifuge was pre‐cooled to 4 °C and centrifuged at 12 000 rpm for 15 min. The supernatant was collected to obtain the protein.

For each group of 3 samples, the protein concentration was determined by the BCA Protein Assay Kit (Beyotime, Shanghai, China). A total of 100 µg of protein was aspirated from each group. Five times the volume of acetone/ethanol/formic acid (50:50:0.1, v/v) was added, and the mixture was thoroughly mixed before allowing the protein to precipitate overnight at −20 °C. After precipitation, samples were centrifuged at 15 000 rpm for 30 min, and the supernatant was discarded. The precipitate was washed twice with acetone. Then, 6 m guanidine hydrochloride solution (soluble in ddH_2_O, 100 µL) was added, and the precipitate was reconstituted by shaking with a tabletop shaker for 30 min (28.65 g for 50 mL). Add 1 m DTT solution (2 µL) and incubate at 60 °C for 1 h. Then, add 1 M IAA solution (10 µL) and incubate at room temperature, protecting from light for 40 min. Transfer the mixture to a 10 kD ultrafiltration tube and centrifuge at 4 °C at 12 000 rpm for 20 min. Wash with NH_4_HCO_3_ solution for 3 times. Add NH_4_HCO_3_ solution (100 µL), followed by Trypsin (trypsin: protein = 1: 50, 100 µg protein add 2 µg trypsin), and replace the collection tube. Perform enzymatic digestion at 37 °C for 16 h. After digestion, centrifuge at 12 000 rpm for 20 min. Add NH_4_HCO_3_ (50 µL) to the ultrafiltration tube and centrifuge for another 20 min. Collect the peptide solution at the bottom of the tube. The collected peptide solution was desalted on a C_18_ column. The eluate was freeze‐dried under vacuum and then dissolved in 0.1% formic acid in water (30 µL). Finally, the sample was analyzed using instrumental LC‐MS (Agilent, USA).

### Cell Cycle Analysis

PANC‐1 and KP4 cells were placed in 6 cm dishes and exposed to designated concentrations of ZQJ29 (1 µm) for 24 h before trypsinization. After centrifugation, the cells were secured and preserved in ice‐cold 70% ethanol and stored at 4 °C for 24 h. They were then thoroughly washed with PBS multiple times. Next, the fixed cells were incubated in PBS supplemented with RNase and PI solution for 30 min at 37 °C, protected from light exposure. Cell cycle progression was assessed using a flow cytometer. This experimental procedure was systematically conducted in three separate trials, with each trial done in triplicate. Cell cycle kits were obtained from Beyotime (Shanghai, China).

### Cell Death Manner Assay

To investigate the mechanism of ZQJ29 in pancreatic cancer proliferation inhibition, the manners of cell death were analyzed. The cell apoptosis inhibitor Z‐VAD‐FMK, cell autophagy inhibitor 3‐methyladenine, cell necrosis inhibitor necrostatin‐1, cell ferroptosis inhibitor Ferrostatin‐1, and cell ferroptosis agonist Erastin were purchased the reagent company, company an (MCE, USA) and stored at −20 °C. PANC‐1 and KP4 cells were seeded with a density of 2000 cells/100 µL well^−1^ into 96 well plates. After 24 h, 10 µm Z‐VAD‐FMK, 5 mm 3‐methyladenine, 10 µm necrostatin‐1, 10 µm Ferrostatin‐1, and 10 µm Erastin were added and co‐incubated with ZQJ29 (1 µm), respectively, for 24 h. Then, 10 µL CCK‐8 was added and incubated at 37 °C for an additional 1 h, the absorbance was measured at 450 nm. The percentage of viability was calculated.

### Measurement of Irons

The iron colorimetric assay kit (Elabscience, Wuhan, China) was used to detect total intracellular iron. PANC‐1 and KP4 cells were cultured in 6‐well plates with a density of 2 × 10^5^ cells per well. After incubation with ZQJ29 (0, 1, 2.5, and 5 µm) for 24 h, the cells were harvested and lysed. The supernatant was collected by centrifugation for iron level detection.

### MDA Assay

Total MDA levels was measured using an MDA assay kit (Jiancheng, Nanjing, China). PANC‐1 and KP4 cells were cultured in 6‐well plates a density of 2 × 10^5^ cells per well. Following incubation with ZQJ29 (0, 1, 2.5, and 5 µm) for 24 h, the cells were harvested, resuspended in PBS, and lysed by sonication. The supernatant was collected by centrifugation for MDA level detection.

### GSH Assay

Total GSH levels were measured using a GSH assay kit (Jiancheng, Nanjing, China). PANC‐1 and KP4 cells were cultured in 6‐well plates with a density of 2 × 10^5^ cells per well. Following incubation with ZQJ29 (0, 1, 2.5 and 5 µm) for 48 h, cells were harvested, resuspended in PBS, and lysed by sonication. The supernatant was collected by centrifugation for GSH level detection.

### Measurement of Lipid ROS

The lipid ROS levels were detected using the lipid peroxidation senso ROS Assay Kit (Beyotime, Shanghai, China). PANC‐1 and KP4 cells were cultured in 24‐well plates with a density of 2 × 10^5^ cells per well. After incubation with varying concentrations of ZQJ29 (0, 1, 2.5, and 5 µm) for 24 h, the cells were gently washed with PBS and then incubated with the reactive oxygen species positive control (Rosup, 10 µm) for 30 min at 37 °C. Subsequently, the reactive oxygen probe DCFH‐DA (10 µm) was loaded and incubated at 37 °C in the dark for 30 min. Fluorescence images were visualized using the Cytation5 (BioTek, America) instrument.

### Mitochondrial Membrane Potential Assay

Mitochondrial membrane potential assay kits with JC‐1 (Beyotime, Shanghai, China) were used to measure the alteration of mitochondrial membrane potential (ΔΨm). After being treated with different concentrations of ZQJ29 (0, 1, 2.5, and 5 µm) for 24 h, cells were treated with the positive control reagent CCCP (10 µm) for 30 min at 37 °C. The cells were rinsed once with PBS, and the JC‐1 staining working solution was added for 30 min at 37 °C in the dark. Afterward, the cells were washed twice with 1 × JC‐1 staining buffer. Fluorescent images of the JC‐1‐stained cells were captured with a fluorescence microscope Cytation5 (BioTek, America).

### Western Blot

For western blotting analysis, ZQJ29‐treated cells were lysed with RIPA Lysis Buffer (Yeasen, Shanghai, China) containing protease and phosphatase inhibitors (Yeasen, Shanghai, China) on ice. The cell lysates were quantified using the BCA Protein Assay Kit (Beyotime, Shanghai, China). Samples were subjected to SDS‐PAGE and transferred to PVDF membranes (Beyotime, Shanghai, China). The membranes were blocked with 5% nonfat milk in TBST (Buffered Saline with 0.1% Tween 20) for 1 h, followed by overnight incubation at 4 °C with primary antibodies. After washing the membranes three times with TBST, they were incubated with the appropriate secondary antibodies for 2 h at room temperature. Following another three times with TBST, the bands on the membranes were detected using the ECL‐Plus Western Blotting Detection System (Yeasen, Shanghai, China).

### Immunofluorescence

PARP1 expression in the cell nucleus was detected using immunofluorescence. The cells were seeded in a 12‐well plate and treated with ZQJ29 (0, 1, 2.5, and 5 µm) after complete adhesion. After a specific duration of treatment, fixation, blocking, and antibody incubation were performed, and images were captured using a Cytation 5‐cell imaging microplate detection system.

### siRNA Transfection and Knockdown of PARP1

A short interfering RNA (siRNA) targeting human PARP1 was designed and synthesized by GenePharma (Shanghai, China). The sequences of the siRNAs are detailed in Table S5 (Supporting Information). PANC‐1 and KP4 Cells were cultured in six‐well plates until they reached 70% confluence. The cells were then transfected with 50 nm siRNA‐PARP1 or a negative control with Lipofectamine 2000 (Thermo Fisher, USA) for 24 h, according to the manufacturer's instructions.

### SPR Analysis

The interaction between ZQJ29 and PARP1 was assessed by SPR with the Biacore S200 system at 25 °C (Cytiva). Recombinant human PARP1 protein (Sino Biological, 11040‐H08B) was immobilized on an activated carboxymethylated 5 (CM5) sensor chip using the amine coupling method. Gradient concentrations of 3 were injected at a flow rate of 40 µL min^−1^ in a running buffer [5% (v/v) DMSO in PBS·P]. The results were analyzed with the Biacore evaluation software (S200 version). Data were fitted to the Steady State Affinity model, and kinetic parameters were derived.

### DARTS Analysis

Total cellular proteins from PANC‐1 and KP4 were extracted using M‐PER lysis buffer (Thermo Fisher, USA). Aliquots of 50 µg of protein were taken from the lysate and placed into each 1.5 mL tube. Different concentrations of ZQJ29 were added to some tubes, while control tubes received an equal amount of DMSO. The mixtures were incubated at room temperature for 1 h. After incubation, Pronase was added to the mixture of cellular proteins at a ratio of Pronase: protein = 1: 3000 for digestion at room temperature for 15 min. Following digestion, 5 × loading buffer was added, and the samples were heated at 100 °C for 10 min before being used for western blot analysis.

### CETSA Analysis

PANC‐1 and KP4 cells were treated with M‐PER lysis buffer to lyse the cells. The resulting protein extracts were divided into two parts. One part was treated with 50 µm ZQJ29, while the other received an equal amount of DMSO. Both mixtures were incubated on ice for 1 h. The samples were then divided into 100 µL PCR tubes and exposed to a range of temperatures starting at 45 °C. After cooling and centrifugation, 5 × SDS loading buffers were added to the supernatant. The samples were then heated to 95 °C for 10 min and analyzed SDS‐PAGE followed by Western blotting.

### Molecular Docking

Molecular docking was conducted to verify the binding site between compound ZQJ29 and key residues. AutoDock Vina, a program that employs a semi‐flexible docking method, was utilized for this study. The small molecular ZQJ29 was designated as the ligand, while the PARP1 protein (ID: 7KNN) served as the receptor. PyMOL (version 2.2.0) software (https://pymol.org/) was used to separate the original ligand and protein structures, as well as to perform dehydration and remove organic matter. The structure format of the PARP1 protein and small molecule ligand were converted from “.PDB” to “.PDBQT” for further docking in AutodockTools. After docking with Vina, the binding scores for the interaction between the PARP1 protein and the small molecule ZQJ29 were calculated. Interaction analysis and visualization of both 3D and 2D representations were performed using PyMOL.

### Statistical Analysis

All data were presented as mean ± SD and were statistically analyzed and graphed using GraphPad Prism software (version 9.0.0). An independent samples t‐test was employed for two‐by‐two comparisons, while one‐way analysis of variance (ANOVA) was used for comparisons of multiple datasets. Tumor growth data from the mice were analyzed using two‐way ANOVA with repeated measures. Data quantification was performed using ImageJ‐win64.

## Conflict of Interest

The authors declare no conflict of interest.

## Supporting information



Supporting Information

## Data Availability

The data that support the findings of this study are available from the corresponding author upon reasonable request.
