# Supplementary material for: Novel Cyano‐Artemisinin Dimer ZQJ29 Targets PARP1 to Induce Ferroptosis in Pancreatic Cancer Treatment
Source: Adv Sci (Weinh). 2025 May 19;12(29):e01935. doi: 10.1002/advs.202501935 (PMC12362764; doi:10.1002/advs.202501935)
Supplement: Supplementary file 1 — Supporting Information [file ADVS-12-e01935-s001.pdf]

ADVANCED SCIENCE
Open Access

Supporting Information

for *Adv. Sci.*, DOI 10.1002/advs.202501935

Novel Cyano-Artemisinin Dimer ZQJ29 Targets PARP1 to Induce Ferroptosis in Pancreatic Cancer Treatment

Jianping Chen, Lingyun Yue, Yanna Pan, Bingying Jiang, Junfeng Wan, Haixia Lin, Fujiang Guo, Huiyu Li, Yajuan Li* and Qingjie Zhao**

Supporting Information

Novel Cyano-Artemisinin Dimer ZQJ29 Targets PARP1 to Induce Ferroptosis in Pancreatic Cancer Treatment

Jianping Chen, Lingyun Yue, Yanna Pan, Bingying Jiang, Junfeng Wan, Haixia Lin, Fujiang Guo, Huiyu Li, Yajuan Li,* and Qingjie Zhao**

J. Chen, L. Yue, Y. Pan, B. Jiang, Q. Zhao

State Key Laboratory of Discovery and Utilization of Functional Components in Traditional Chinese Medicine, Shanghai Frontiers Science Center for TCM Chemical Biology, Innovation Research Institute of Traditional Chinese Medicine, Shanghai University of Traditional Chinese Medicine

E-mail: zhaoqingjie@shutcm.edu.cn

J. Chen, Y. Li

Institute of Interdisciplinary Integrative Medicine Research,Shanghai University of Traditional Chinese Medicine, Shanghai 201203, China

E-mail: liyajuan9@sina.cn

L. Yue, H. Lin

Department of Chemistry, College of Sciences, Shanghai University

Shanghai 200444, China

B. Jiang, F. Guo

School of Pharmacy, Shanghai University of Traditional Chinese Medicine

Shanghai 201203, China

J. Wan, H. Li

College of Mathematics and Physics, Shanghai University of Electric Power

Shanghai 201306, China

E-mail: huiyuli@shiep.edu.cn

Table S1. ^1H NMR (400 MHz) and ^{13}C NMR (100 MHz) spectroscopic data for compounds ZQJ29 (δ in ppm, J in Hz).

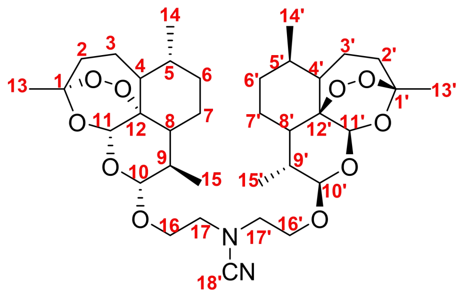

no	δ_C (type)	δ_H (type, J in Hz)
1	104.33, C	
2	36.54, CH_2	2.40–2.32, m
3a	24.80, CH_2	1.91–1.85, m
3b		2.06-2.00, m
4	52.68, CH	1.27–1.22, m
5	37.50, CH	1.38–1.35, m
6a	34.72, CH_2	1.67–1.62, m
6b		0.95-0.93, m,overlap
7	24.54, CH_2	1.80–1.75, m
8	44.44, CH	1.53-1.47, m
9	30.87, CH	2.69–2.62, m
10	102.63, CH	4.82, d, (3.6)
11	88.16, CH	5.43, s
12	81.15, C	
13	26.26, CH_3	1.42, s
14	20.50, CH_3	0.95-0.93,m
15	13.15, CH_3	0.95-0.93,m
16a	65.73, CH_2	4.05–4.00, m
16b		3.61–3.56, m
17	51.81, CH_2	3.33–2.22, m
18	117.30, C	

Table S2. IC_{50} (μM) of DHA, SM1044, ZQJ29, and OXA at 72 h against different cell lines. Data are average of 3 parallel test.

Cell lines	ZQJ29	SM1044	DHA	DDP	OXA
PANC-1	0.120±0.030	5.954±0.501	＞10	＞10	＞10
KP4	0.885±0.106	2.995±0.799	＞10	＞10	＞10
A549	＞10	3.445±1.761	＞10	＞10	2.470±0.410
H1975	2.190±0.113	6.880±0.014	＞10	＞10	3.490±0.552
MCF	0.759±0.311	1.335±0.303	＞10	0.036±0.028	0.196±0.087
HeLa	1.283±0.289	2.285±0.429	＞10	0.282±0.110	0.510±0.121

HCT116	1.018±0.500	4.983±2.362	＞10	0.071±0.042	0.103±0.026
JHH7	0.791±0.305	2.129±0.573	＞10	＞10	7.147±2.599
HEK-293T	＞10	＞10	＞10	＞10	＞10
LX2	＞10	＞10	＞10	＞10	2.460± 0.560
THLE-2	＞10	＞10	8.660±0.714	1.461±0.984	0.777±0.188
HPDE6-C7	＞10	＞10	＞10	＞10	2.704±0.567

Tables S3. Pharmacokinetic parameters of ZQJ29 after i.g. administration (n = 6).

Compounds	Dose(mg/kg)	$t_{1/2}$ （h）	C_{max} （μg/L）	AUC_{0-t} （hr*ng/mL）	MRT_{0-t} （h）
ZQJ29	50-i.g.	1.58± 0.26	11.80± 6.05	40.00± 16.30	2.74± 0.46

Tables S4. Antibodies used in this study.

Antibody	Source	Catalog No.
Anti-PARP1	Proteintech	66520-1-Ig
Anti-TP53	Proteintech	10442-1-AP
Anti-SLC7A11	Proteintech	26864-1-AP
Anti-GPX4	Proteintech	67763-1-Ig
GAPDH	Proteintech	60004-1-Ig
Anti-Ki67	Abcam	Ab15580
Anti-PCNA	Cell Signaling Technology	2586

Tables S5. Sequences of siRNAs used in this study.

Name	siRNAs
siRNA-PARP1-1	CGCCCAUGUUUGAUGGAAATT
	UUUCCAUCAAACAUGGGCGTT
siRNA-PARP1-2	GGUGAUCGGUAGCAACAAATT
	UUUGUUGCUACCGAUCACCTT

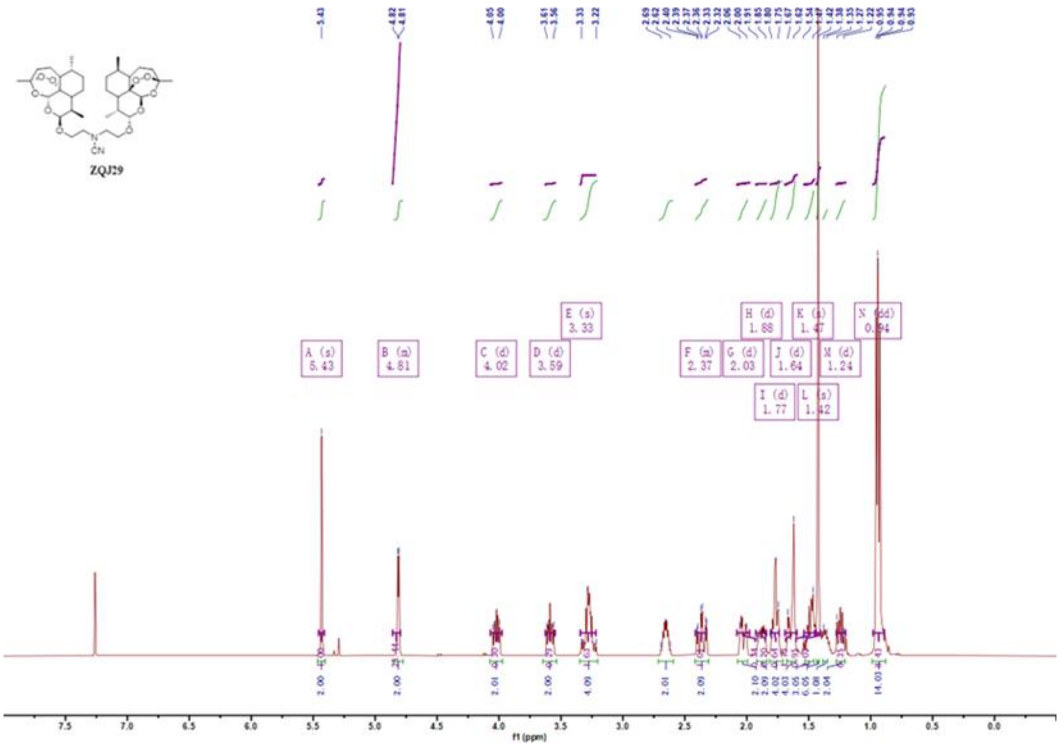

Figure S1. The ^{1}H NMR date of ZQJ29

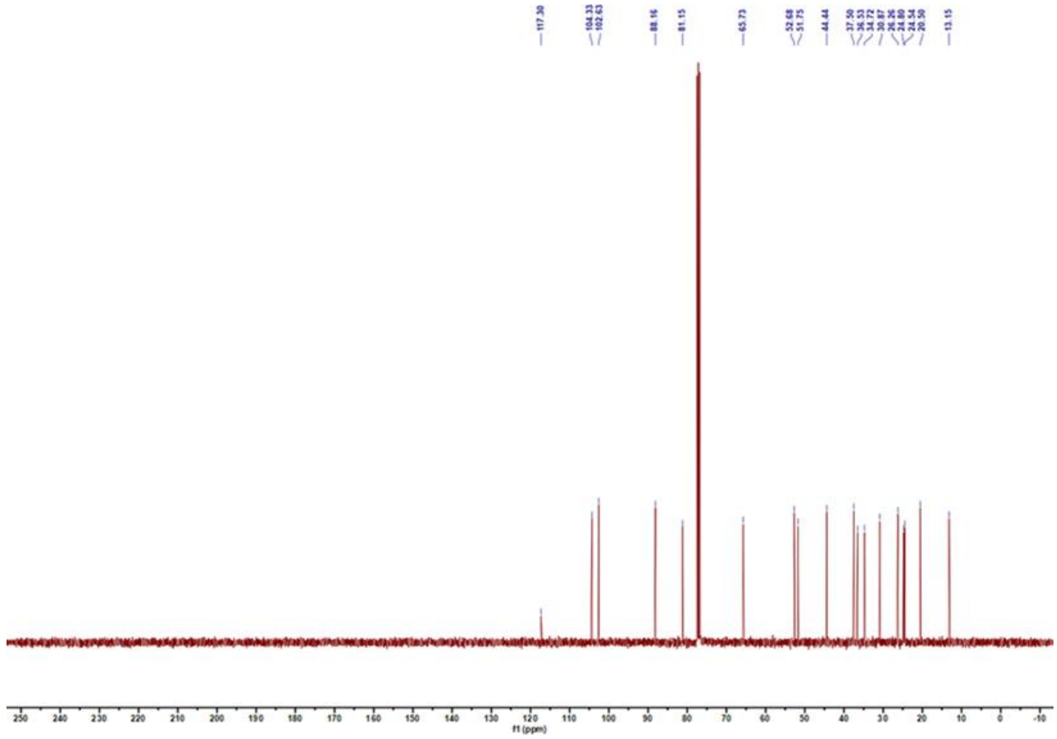

Figure S2. The ^{13}CNMR date of ZQJ29.

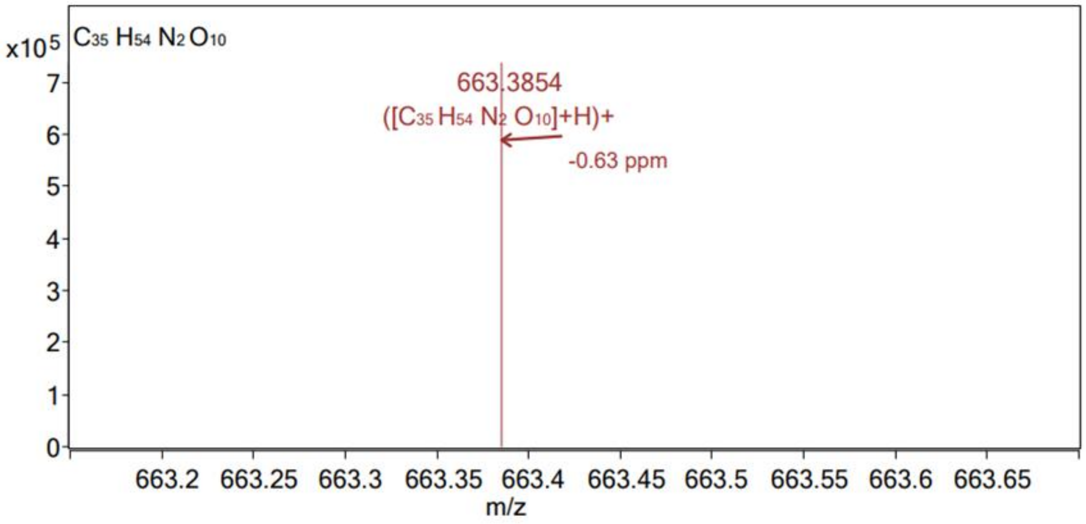

Figure S3. The ESI-HRMS spectra of ZQJ29.

色谱图

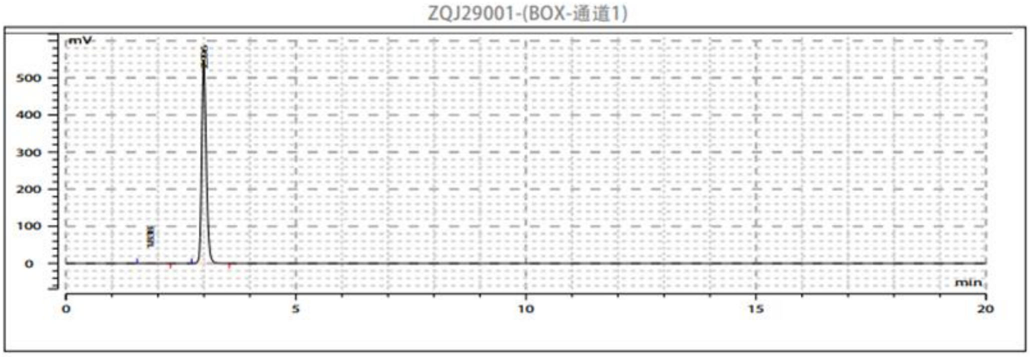

峰列表

ZQJ29001-(BOX-通道1)

No	名称	保留时间 (min)	峰面积 (mV*s)	峰宽 (min)	半峰宽 (min)	峰高 (mV)	面积百分比 (%)	峰类型	峰纯度	PDA 匹配名称
1	N.A.	1.838	2.276	0.158	0.096	0.355	0.060	BB*	0	
2	N.A.	2.996	3803.154	0.176	0.104	549.819	99.940	BB	0	
3	总计									

Figure S4. HPLC profiles of ZQJ29.

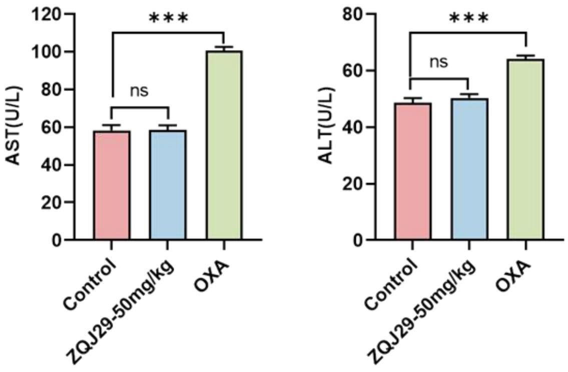

Figure S5. Blood routine and blood biochemical index of the mice treated with ZQJ29 at the dose of 50 mg/kg or OXA. Alanine aminotransferase（ALT), Aspartate aminotransferase (AST). Data

represent mean ± SD (n = 6).

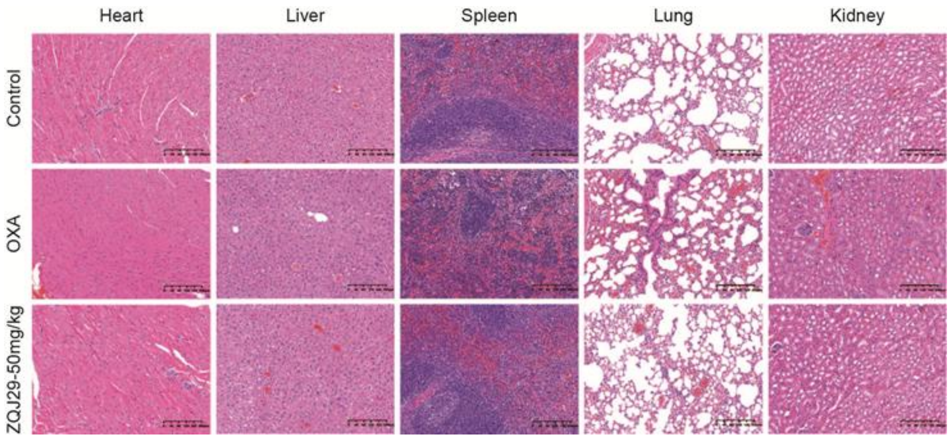

Figure S6. H&E staining images of major organs of BALB/c nude mice.

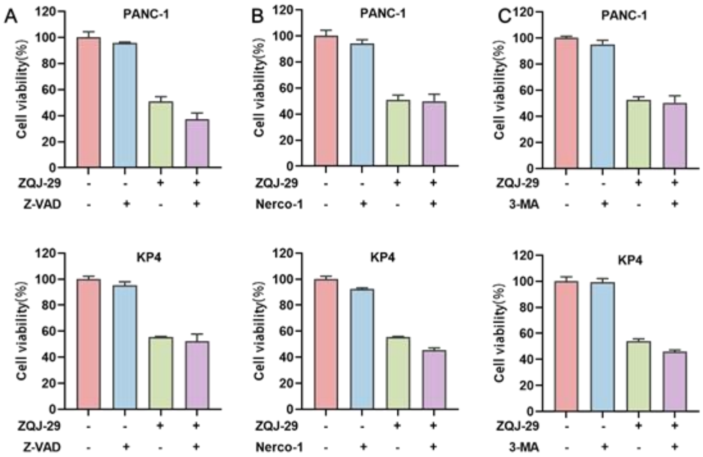

Figure S7. KP4 and PANC-1 cell death mode assay after ZQJ29 treatment. (A) The percentage of cell viability after the combination of ZQJ29 (0.5 μM) with Z-VAD-FMK (10 μM) for 24 h. (B) The percentage of cell viability after the combination of ZQJ29 (0.5 μM) with necrosis inhibitor necrostatin-1 (10 μM) for 24 h. (C) The percentage of cell viability after the combination of ZQJ29 (0.5 μM) with 3-methyladenine (5 mM) for 24 h. (D) The percentage of cell viability after the combination of ZQJ29 (0.5 μM) with ferroptosis inhibitors Ferrostatin-1 (10μM). Analysis results represented mean ± SD,**P* < 0.05, ***P* < 0.01, ****P* < 0.001.

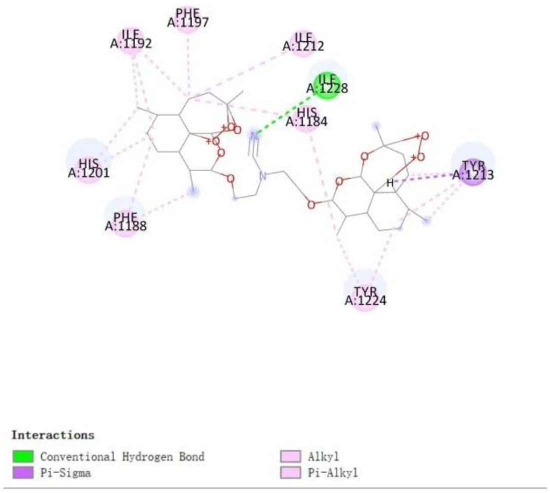

Binding Energy:-13.19 kcal/mol

Figure S8. Molecular docking diagram of interaction between PARP1 and ZQJ29.
